# Impact and Tensile Properties of Polyester Nanocomposites Reinforced with Conifer Fiber Cellulose Nanocrystal: A Previous Study Extension

**DOI:** 10.3390/polym13111878

**Published:** 2021-06-05

**Authors:** Grazielle da Silva Maradini, Michel Picanço Oliveira, Lilian Gasparelli Carreira, Damaris Guimarães, Demetrius Profeti, Ananias Francisco Dias Júnior, Walter Torezani Neto Boschetti, Bárbara Ferreira de Oliveira, Artur Camposo Pereira, Sergio Neves Monteiro

**Affiliations:** 1 Forest and Wood Sciences Department, Federal University of Espírito Santo, Jeronimo Monteiro 29550-000, Brazil; graziellemaradini@gmail.com (G.d.S.M.); michelpicanco@gmail.com (M.P.O.); ananiasjuniorr@gmail.com (A.F.D.J.); 2 Rural Engineering Department, Federal University of Espírito Santo, Alto Universitário, sn., Alegre 29500-000, Brazil; lcarreira83@gmail.com (L.G.C.); guimaraes.damaris@yahoo.com.br (D.G.); 3 Chemistry and Physics Department, Federal University of Espírito Santo, Alto Universitário, sn., Alegre 29500-000, Brazil; demetrius.profeti@ufes.br; 4 Department of Forest Engineering, Federal University of Viçosa, Viçosa 36570-900, Brazil; walterboschetti@gmail.com; 5 Advanced Materials Department, Northern Fluminense State University Campos dos Goytacazes, Campos dos Goytacazes 28013-602, Brazil; barbara.fo@gmail.com; 6 Military Institute of Engineering—IME, Materials Science Program, Praça General Tibúrcio 80, Urca, Rio de Janeiro 22290-270, Brazil; camposo.artur@gmail.com

**Keywords:** nanocomposite, chemical analysis, mechanical behavior, polyester, cellulose nanocrystal

## Abstract

In a recent paper, novel polyester nanocomposites reinforced with up to 3 wt% of cellulose nanocrystals (CNCs) extracted from conifer fiber were characterized for their crystallinity index, water absorption, and flexural and thermal resistance. The use of this novel class of nanocomposites as a possible substitute for conventional glass fiber composites (fiberglass) was then suggested, especially for the 1 and 2 wt% CNC composites due to promising bending, density, and water absorption results. However, for effective engineering applications requiring impact and tensile performance, the corresponding properties need to be evaluated. Therefore, this extension of the previous work presents additional results on Izod and tensile tests of 1 and 2 wt% CNC-reinforced polyester composites, together with a comparative cost analysis with fiberglass. The chemical effect caused by incorporation of CNCs into polyester was also investigated by FTIR. In comparison to the neat polyester, the Izod impact energy increased 50% and 16% for the 1 and 2 wt% composites, respectively. On the other hand, the tensile strength and Young’s modulus remained constant within the ANOVA statistical analysis. FTIR analysis failed to reveal any chemical modification caused by up to 2 wt% CNC incorporation. The present impact and tensile results corroborate the promising substitution of a polyester composite reinforced with very low amount of CNCs for common fiberglass in engineering application.

## 1. Introduction

**Figure 1:**
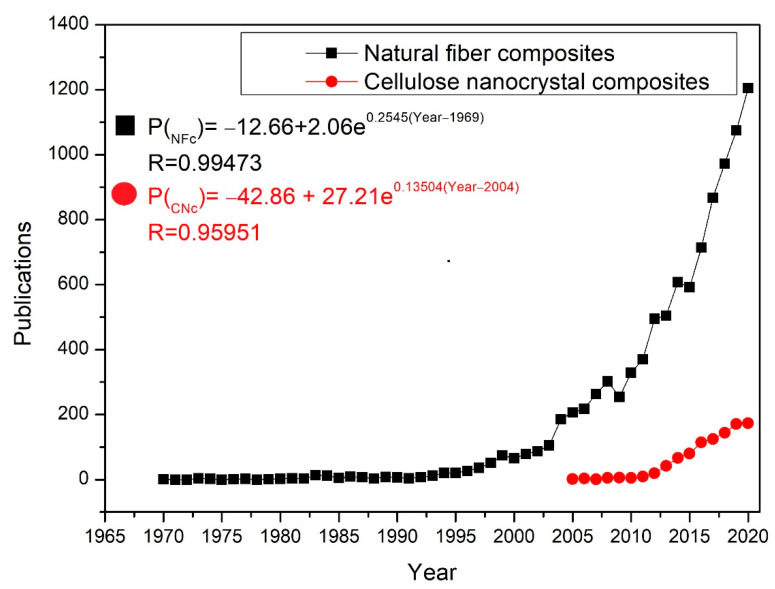
Correlations of the publications by the year of the natural fiber composites and cellulose nanocrystal composites.

This century is experiencing an exponential increase in both the research works and industrial application of natural lignocellulosic fiber (NLF)-reinforced polymer matrix composites, as illustrated in the black squares curve in Figure 1 drawn by Luz et al. [1] from the Scopus database [2]. Particularly in recent decades, many researches have been carried out on composites reinforced with NFLs produced with fibers in different orientations, usually in amounts higher than 10% as well as their respective fabrics [3,4,5,6]. Another recently emerging potential use of NLF, indicated by the red circles curve in Figure 1, is through the extraction of its cellulose nanocrystals (CNCs) for the reinforcement of nanocomposites [7,8,9,10,11,12,13,14,15].

In fact, CNCs display outstanding properties such as surface to volume ratios of ~100, relatively low densities, and high mechanical strength [14,15]. According to Kargarzadeh et al. [14], CNCs can be easily modified as well as readily available, renewable, and biodegradable. They are rod-like particles with transverse dimensions as small as 3–30 nm [16] and lengths ranging between 100 and 1000 nm. In contrast to NLF, the incorporation of CNCs into polymer matrices usually requires low percentages (<10 wt%) to effectively reinforce nanocomposites [7,8,9,10,11,12,13,14,15] for a wide range of applications, including food packing and biomedical devices [8,9,10,11,12,13,14,15,16]. This has motivated the exponential rise of publications related to CNC nanocomposites in the past decade, as illustrated by the rede curve obtained from Scopus database in Figure 1.

Different NLFs, such as kenaf [14], sugar palm [17], soft and hard wood mixtures [18], rice husk [19], banana [20], sisal [21], and tunicin [22], have been used to isolate CNCs. Among the polymer matrices reinforced by these CNCs, unsaturated polyester is one of the thermoset resins that is most used for high-performance nanocomposites due to its room temperature (RT) cure associated with elevated strength, water resistance, and transparency properties [14]. However, the relatively low toughness of polyester limits its application as an engineering component subjected to impact. In contrast, polyester composites reinforced with more than 10 wt% of glass fiber, also called fiberglass, present an enhanced toughness that allows for applications in many industrial sectors, including aerospace and high-performance sports equipment, that require resistance to impact load [23,24,25].

A relevant point is the dispersion of hydrophilic CNCs into a hydrophobic polyester matrix. To prevent the aggregation of CNCs, surface modification by chemical coupling agents might not only improve dispersion but also enhance nanocomposite mechanical properties [26]. In the present work, a styrene monomer was used as coupling agent between a polyester matrix and CNCs following similar conditions used in a previous publication [27]. In that publication, polyester nanocomposites reinforced with 1, 2, and 3 wt% of CNCs extracted from conifer wood fiber were characterized for the crystallinity index and water absorption, as well as flexural and thermal resistance. The use of these novel nanocomposites was indicated as a possible substitute for conventional fiberglass in engineering applications. In particular, promising bending and thermal resistance preliminary results were found for the 1 and 2 wt% CNC nanocomposites. However, for industrial applications requiring impact and standard tensile performance, other specific properties need to be evaluated together with a cost-effectiveness comparison with fiberglass. Thus, this work presents additional results on Izod and tensile tests, as well as a comparative cost analysis for the same [27] previously investigated 1 and 2 wt% CNC-reinforced polyester composites. Fourier transform infrared spectroscopy as used to investigate possible chemical effects caused by CNC incorporation into polyester.

## 2. Materials and Methods

### 2.1. Materials

The same materials previously investigated in [27] were used in this work. Briefly, unsaturated polyester resin (average molecular weight Mn = 9 × 10³ g/mol, a butanox catalyst (Mt 50), and a styrene monomer (coupling agent) were all supplied by Redelease, Brazil. Conifer fiber CNCs were provided by the Development Center of the University of Maine, Orono, ME, USA, with transverse dimensions of 3.0 ± 0.5 nm and a length of 190 ± 15 nm.

### 2.2. Processing of Nanocomposites

**Figure 2:**
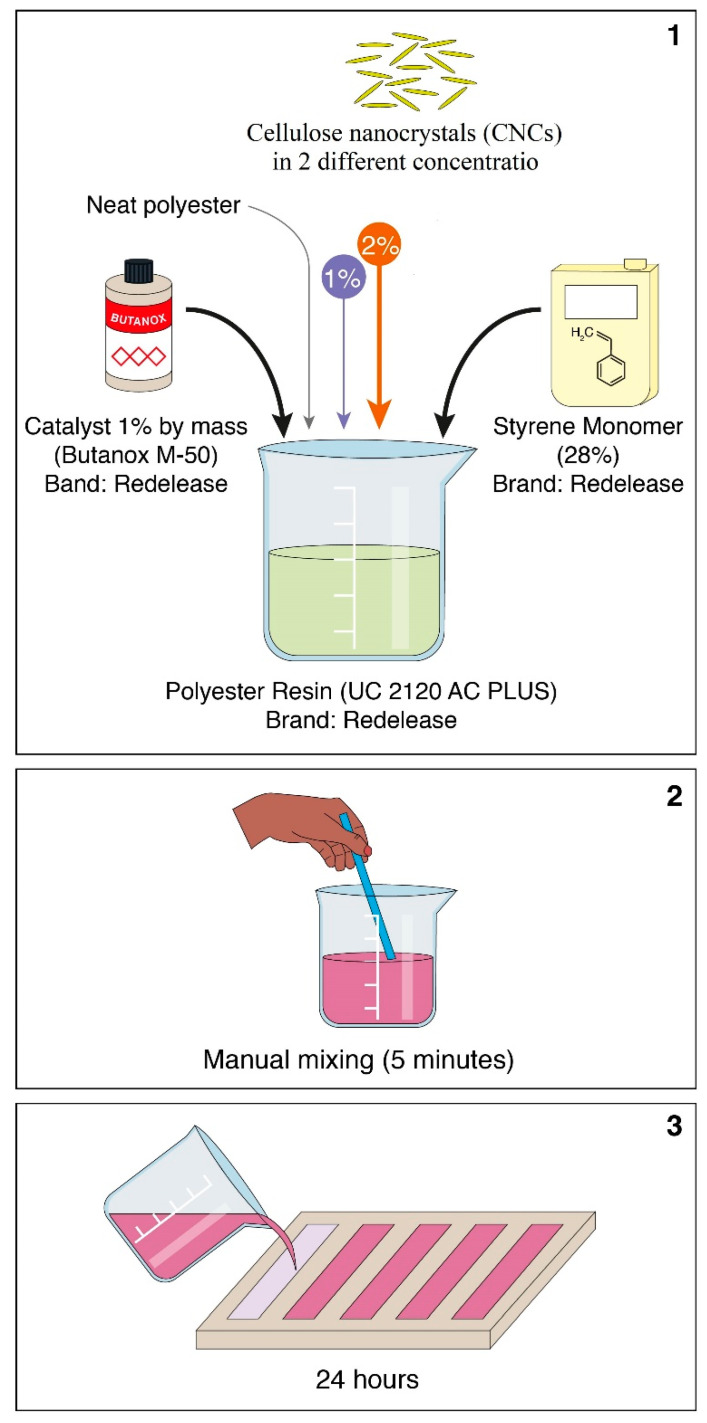
Schematic fabrication of unsaturated polyester composites reinforced with CNCs.

Similar procedures described in [27] were caried out when processing the nanocomposites. Briefly, separate amounts of 1 and 2 wt% of CNCs were initially mixed with the styrene monomer and still fluid polyester/1% butanox. The mixture was then poured into silicone molds with shapes and dimensions defined by the D256 [28] and D3039 [29] ASTM standards for Izod and tensile specimens, respectively. Four specimens were fabricated for each type of test and distinct amounts of CNCs, and they were finally cured at ambient temperature and pressure for 24 h. The nanocomposites presented densification parameters associated with void volume fractions of 5.6, 6.4, and 7.0% for 0 (neat polyester), 1, and 2 wt% CNC, respectively. Figure 2 schematically shows the composite fabrication process.

### 2.3. Fourier Transform Infrared Analysis (FTIR)

FTIR spectra were recorded at RT in a Bruker spectrometer, model Tensor 27 (Leipzig, Germany), by the attenuated total reflectance (ATR) technique. The powder samples were scanned in the range of 4000–600 cm^−1^ with a resolution of 4 cm^−1^. In this analysis, 32 scans were collected for each FTIR spectrum.

### 2.4. Izod Impact Test

**Figure 3:**
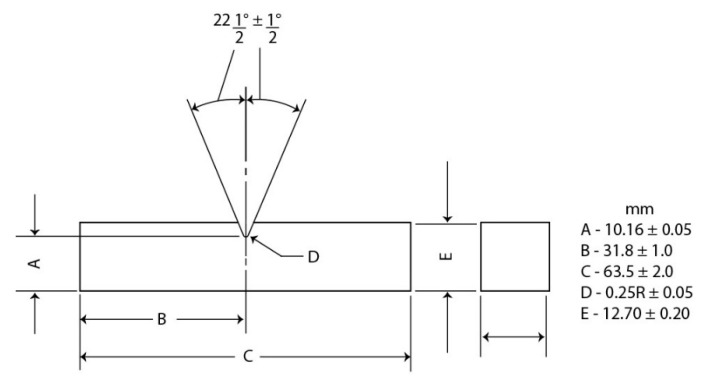
Schematic of dimensions of Izod-type test specimen according to the standard.

Izod tests were carried out at RT for each CNC composition in a Pantech Instruments (São Paulo, Brazil), using a 11 J hammer pendulum. Prismatic standard [28] notched specimens with a depth of 2.54 mm and an angle of 45° were fabricated using a Notchvas model CEAST carver. Four samples for each percentage of CNCs with dimensions of 62.5 × 12.7 × 10 mm were produced. Figure 3 shows schematics the specimens of the Izod tests.

### 2.5. Tensile Test

**Figure 4:**
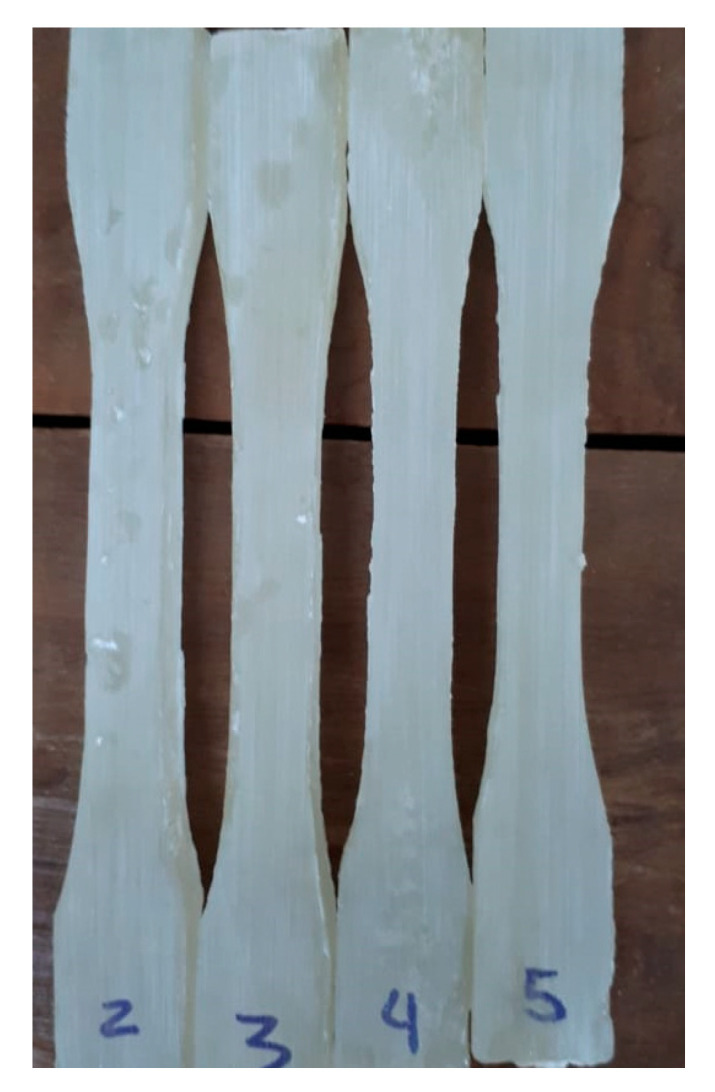
Specimens nanocomposites with 1 wt% of CNCs for tensile test.

Tensile tests were conducted at RT on four standard [29] specimens for each CNC composition in a model DL 10.000 EMIC universal machine, (São José dos Pinhais, Brazil), operating with cross-head speed of 1 mm/min until the specimen ruptured using a 1 kN load cell. Typical specimens prepared for the tensile tests are shown in Figure 4.

### 2.6. Statistical Analysis

The analysis of variance (ANOVA) was applied to test the hypothesis of equality, and a lower significant difference Tukey test was applied for the Izod and Young’s modulus and ultimate tensile strength, both with 95% confidence level, results.

## 3. Results and Discussion

### 3.1. Fourier Transform Infrared Analysis Spectroscopy (FTIR)

**Figure 5:**
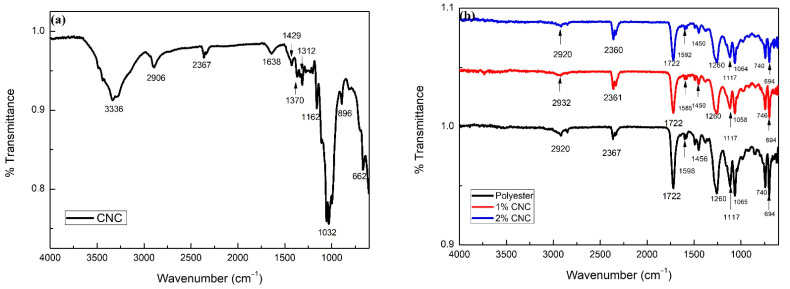
FTIR spectra of (**a**) plain CNC and (**b**) polyester nanocomposites, as well as neat polyester.

Figure 5 shows the FTIR spectra of plain CNC (Figure 5a) and polyester nanocomposites reinforced with 1 and 2 wt% of CNCs and neat polyester (Figure 5b).

In Figure 5a, the wide band at 3336 cm^−1^ is related to the O–H group vibration with strong intramolecular H bonds, which may have been associated with the alcohols, extracts, and carboxylic acids that make up cellulose. In some cases, due to the crystalline or hindrance steric structure, the hydroxyl group was not hydrogen-bonded. For this reason, a resultant weak band appeared between 3500 and 3300 cm^−1^, as observed in the cellulose FTIR spectrum of Figure 5a. Moreover, the weak intensity of the OH band is usually found when an ATR accessory is used for the IR measurements of polymers. A typical cellulose FTIR/ATR spectrum was shown in the study of Larkin [30], which corroborated the CNC profile obtained in our work. It is important to mention that these CNC nanocomposites showed an irrelevant water absorption of only 0.2%, as reported in our previous work [27]. The 2906 cm^−1^ band corresponds to the C–H bonds, which are characteristic of organic molecules found in natural components. The weak band around 2367 cm^−1^ refers to the CO_2_ present in the atmosphere during the analysis. The band at 1638 cm^−1^ is related to OH groups and water molecules absorbed onto the CNC surface [31,32,33]. The bands at 1429 and 1312 cm^−1^ can be attributed to symmetric angular strain in the plane of the CH_2_ group and symmetrical angular strain in the plane of the CH_2_ groups, respectively. The band around 1370 cm^−1^ can be attributed to the C–H strain. The band at 1162 cm^−1^ corresponds to the angular deformation of the C–O connections of the esters existing in the CNC. The bands at 1032 and 896 cm^−1^ indicate the purity of the crystalline cellulose. The band at 1032 cm^−1^ is attributed to the vibrations of the C–O. The band at 896 cm^−1^ is related to the axial deformation of the C–O–C bonds and β-glycosidic bonds present between the cellulose glucose groups [34,35,36]. The band around 662 cm^−1^ may be assigned to the -CH- bonding of aromatic groups [32].

It is worth noting that the CNC spectrum in Figure 5a displays transmittance at 1429, 1162, and 896 cm^−1^, indicating that the nanocellulose produced before acid hydrolysis was in the form of cellulose I, which corresponds to native cellulose [34,35,36,37]. In Figure 5b, it can be observed that the unsaturated polyester had a weak band at 2920 cm^−1^, which can be attributed to the C–H elongation. The weak band around 2367 cm^−1^ refers to the CO_2_ present in the atmosphere during the analysis, and the band at 1456 cm^−1^ is not associated with any functional group present in the unsaturated polyester. The polyester showed important characteristic absorption in the 1722 cm^−1^ band, which represents the carbonyl group, C=O. The bands close to 1598 and 740 cm^−1^ represent the elongation of the aromatic nucleus C=C. This occurred due to the presence of the unsaturated double bond (C=C) in the polyester and refers to the vinyl group present in the styrene monomer. The bands close to 1260 and 1117 cm^−1^ occurred due to stretching vibrations C–O–C connected to the aliphatic and aromatic groups, respectively [38,39]. The bands at 1065 and 694 cm^−1^ can be assigned, respectively, to the aromatic C–H ring in the plane and the aromatic C=C ring in the plane [38].

The FTIR spectra in Figure 5 of polyester nanocomposites reinforced with 1 and 2 wt% of CNCs show absorption bands similar to those of the polyester without reinforcement. This indicates that the incorporation CNCs into the polyester did not significantly change the chemical structure However, there were minor variations in the frequencies of the absorption bands of the nanocomposites in comparison to those of the polyester. These observed changes in the bands may indicate reinforcement load interactions with the matrix [39,40].

### 3.2. Izod Impact Strength

**Figure 6:**
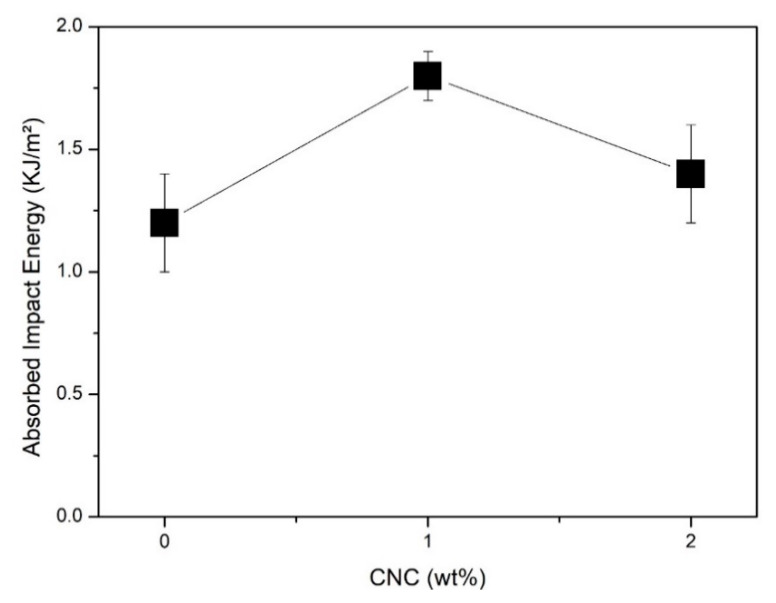
Variation of the Izod absorbed impact energy of unsaturated polyester nanocomposite notched specimens with CNC content.

Figure 6 shows the variation of Izod absorbed impact energy with CNC content in unsaturated polyester matrix. A significant increase of 50% was obtained with the addition of 1 wt% of CNCs into the polyester matrix, which proved a reinforcement effect in impact strength. For the 2 wt% CNC nanocomposites, the average absorber impact energy showed an increase of 16%. However, the relatively higher standard deviations (error bars) did not guarantee a reinforcement effect. An important comparison might be done with the CNC (isolated from kenaf bast fiber)-reinforced unsaturated polyester nanocomposite impact energy results of Kargarzadeh et al. [14]. Though their impact test was performed with unnotched specimens, which gave comparatively higher impact strengths for the polyester matrix, the incorporation of 2 wt% of CNCs increased the impact energy by 14%. Another relevant point discussed by the authors was the fact that the incorporation of inorganic nanoparticles such as 5 wt% Al_2_O_3_ [41] and 2 wt% nonclay [42] into unsaturated polyester improved the impact resistance by 11% and 20%, respectively. Thus, the results presented in Figure 3 corroborate the conclusion of Kargarzadeh et al. [14] that CNC has the potential to be used as an impact modifier for different types of polyester nanocomposites

Regarding these findings in CNC-reinforced polyester nanocomposites, it is worth mentioning that Bindal et al. [23] reported a much lower impact strength of polyester reinforced with 20 wt% of glass fiber. A question that may arise from the results in Figure 6 is the reason for the decrease in impact strength in going from 1 to 2 wt% of CNCs. According to Peng et al. [43], nanocomposite impact strength decreases with increasing additions of CNC, which may be due to the agglomeration of nanoparticles. Therefore, as evidence in Figure 6, there is no need to reinforce a polyester matrix with more than 2 wt% of conifer fiber CNC to obtain a significant impact strength to compete with nanocomposites reinforced with inorganic nanoparticles or fiberglass at cost-effective conditions.

Another question regarding the values and corresponding error bars in Figure 6 is the effective reinforcement caused by the incorporation of CNC into polyester matrix. The ANOVA comparing neat polyester (0 wt%) and the 1 wt% CNC nanocomposite showed that F_cal._ (19.5923) > F_crit_ (5.98737), which indicated that their impact strengths were different with 95% of confidence.

From the Tukey test results, it was possible to affirm with a 95% level of confidence that the impact strength of the 1 wt% CNC nanocomposite was higher not only than that of the neat polyester but also the 2 wt% CNC nanocomposite. Moreover, both neat polyester and the 2 wt% CNC composite had similar impact strengths.

### 3.3. Tensile Test

**Figure 7:**
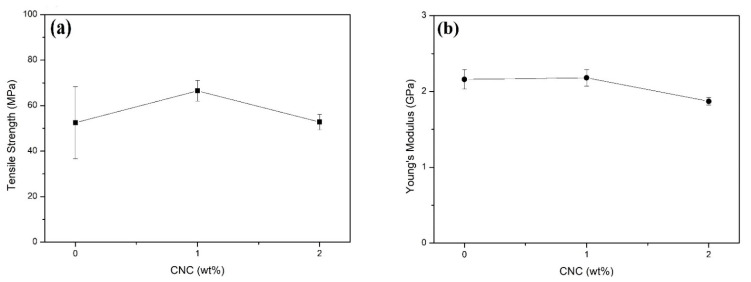
Variation of (**a**) tensile strength and (**b**) Young’s modulus of unsaturated nanocomposites with conifer fiber CNC content.

Figure 7 show the variation of (Figure 7a) tensile strength and (Figure 7b) Young’s modulus with conifer fiber CNC content in unsaturated polyester nanocomposites. In Figure 7a, one can notice a maximum in the average value of the tensile strength with the incorporation of 1 wt% of CNCs. However, due to the relatively higher standard deviation associated with the neat polyester, it is not possible to assert the existence of this maximum.

The ANOVA for the CNCs contents of 0 (neat polyester), 1, and 2 wt% revealed an F_cal_ (2.742) < F_crit_ (4.256), indicating no difference between the corresponding tensile strengths with a 95% level of confidence. In this case, there was no need to perform the Tukey test.

As for the Young’s moduli in Figure 7b, there was no apparent increase with 1 wt% conifer fiber CNC compared to the neat polyester. However, an average decrease of ~15% occurred for the 2 wt% CNC composite as compared to the neat polyester. The ANOVA for the three CNCs contents of 0, 1, and 2 wt% revealed an F_cal_ (11.813) > F_crit_ (4.256), indicating, with a 95% level of confidence, that there was a significant difference between the Young’s moduli in Figure 7b.

In order to verify the origin of the difference detected by the ANOVA, a corresponding Tukey test was performed. From the results, it is possible to affirm with a 95% level of confidence that the Young’s moduli of 0 (neat polyester) and 1 wt% CNC composites were similar but different to that of the 2 wt% CNC composite.

It is worth mentioning that Kargarzadeh et al. [14] found no increase in both tensile strength and Young’s modulus with the incorporation of 2 wt% kenaf fiber CNC compared to an unsaturated polyester matrix. The authors did not investigate 1 wt% CNC nanocomposites.

Based on the results shown in Figure 6 and Figure 7, one could conclude that 1 wt% conifer fiber CNC is able to reinforce a polyester matrix. This finding was corroborated by the results of Cherayil et al. [42], which also suggested that 0.5 wt% conifer fiber CNC might even be a better reinforcement. On the other hand, common polyester composites reinforced with more than 10 wt% of glass fiber (fiberglass) usually display higher tensile strengths than those of the aforementioned results reported in [14,42] and Figure 7 However, Bindal at al. [23] disclosed a tensile strength of 62.2 MPa for a polyester composite with 20 wt% of glass fiber, which was slightly lower than the present value of 65.6 MPa for a 1 wt% conifer fiber CNC polyester nanocomposite.

Despite the difference in mechanical properties, one important factor in deciding to substitute a CNC nanocomposite for fiberglass is the comparative cost of materials. For this purpose, Table 1 presents a preliminary cost analysis of the present polyester nanocomposite with 1 wt% of conifer fiber CNCs and the glass fiber-reinforced polyester composites of Bindal et al. [23].

The results of the cost analysis in Table 1 show that the nanocomposite with 1 wt% of CNCs, despite the very low amount of filler, would always be more expensive due to the greater price of polyester. However, the difference in price is negligible and might never be a factor in engineering application decisions. On the other hand, technical factors such as impact strength (Figure 6), which favor CNC nanocomposites over glass fiber composites [23], might be decisive factors in specifying applications like aerospace components and high-performance sport equipment.

Moreover, it is important to emphasize that glass fiber composites are highly anisotropic, which could limit their applications in certain engineering conditions. In contrast, CNC composites are isotropic, which opens more engineering possibilities.

## 4. Summary and Conclusions

Additional results for unsaturated polyester nanocomposites reinforced with 1 and 2 wt% of CNCs are presented here as an extension of previous work [27]. FTIR spectra revealed that the small incorporation of CNC did not significantly alter the chemical structure of the polyester matrix. The observed changes in bands might indicate filler interaction with the matrix.

- An increase of 50% in the impact strength was obtained for the 1 wt% CNC nanocomposite, which proved to be an effective reinforcement with respect to neat polyester. For the 2 wt% CNC composite, the 16% increase in the average value had standard deviations coinciding with that of polyester and were not found be different via the ANOVA and Tukey test.
- The tensile strength and Young’s modulus of the 1 wt% CNC nanocomposite were not different than those of neat polyester, as supported by the ANOVA and Tukey test. On the other hand, the Young’s Modulus of the 2 wt% nanocomposite decreased by 15% if compared to the neat polyester.
- A preliminary cost analysis found that since polyester is the most expensive precursor, the 1 wt% CNC nanocomposite and glass fiber composites are equally cost-effective.
- Other factors such as impact strength and CNC renewability would favor the present nanocomposite for specific applications in high-performance sport equipment.

## Figures and Tables

**Table 1:** Comparative cost analysis between the present polyester nanocomposite with 1 wt% of conifer fiber CNC and the polyester composites reinforced with glass fiber.

**Precursor Materials**	**Price (US$/Kg)**	**Ref**
Unsaturated Polyester	11.10	[44]
E Glass Fiber	6.90	[45]
Conifer Fiber CNC	8.16	[present work]
**Investigated Materials**	**Calculation**	**Final Cost (US$/Kg)**
1 wt% Conifer fiber CNC/Polyester	0.01 × 8.16 + 0.99 × 11.10	11.07
20 wt% Glass Fiber/Polyester	0.2 × 6.90 + 0.80 × 11.10	10.26
30 wt% Glass Fiber/Polyester	0.3 × 6.90 + 0.70 × 11.10	9.84
40 wt% Glass Fiber/Polyester	0.4 × 6.90 + 0.60 × 11.10	9.42

## Data Availability

The data presented in this study are available on request from the corresponding author.
